# Supplementary material for: Active site specificity profiling datasets of matrix metalloproteinases (MMPs) 1, 2, 3, 7, 8, 9, 12, 13 and 14
Source: Data Brief. 2016 Feb 22;7:299–310. doi: 10.1016/j.dib.2016.02.036 (PMC4777984; doi:10.1016/j.dib.2016.02.036)
Supplement: Supplementary file 10 — Supplementary material [file mmc10.zip › WebPICS_hMMP9_G_1%/P2prime.html]

 

PICS results


|  |  |
| --- | --- |
| **P2prime\_A**  13 in 146 sites   8.9 %    effects > 10 perc. pnts.  (vice-versa in brackets)  P2\_A: 17.8 (12.2)   P1\_G: 24.1 (14.9)   P1prime\_C: 15.6 (18.4) |  |
| **P2prime\_C**  4 in 146 sites   2.7 %    effects > 10 perc. pnts.  (vice-versa in brackets)  P2\_C: 48.6 (97.3) |  |
| **P2prime\_I**  13 in 146 sites   8.9 %    effects > 10 perc. pnts.  (vice-versa in brackets)  P1\_N: 14.9 (16.1)   P1prime\_V: 22.6 (24.4)   P3prime\_N: 19.0 (41.1) |  |
| **P2prime\_K**  18 in 146 sites   12.3 %    effects > 10 perc. pnts.  (vice-versa in brackets)  P2\_K: 14.1 (12.7)   P1\_A: 22.5 (16.9)   P1prime\_Q: 19.6 (29.4) |  |
| **P2prime\_L**  18 in 146 sites   12.3 %    effects > 10 perc. pnts.  (vice-versa in brackets)  P3\_A: 12.7 (10.4)   P1\_S: 11.2 (12.7)   P1prime\_F: 11.2 (25.2) |  |
| **P2prime\_Q**  6 in 146 sites   4.1 %    effects > 10 perc. pnts.  (vice-versa in brackets)  P2\_Y: 13.3 (15.9)   P1\_C: 15.3 (45.9)   P1\_H: 29.9 (35.9)   P1prime\_Q: 41.8 (20.9) |  |
| **P2prime\_R**  17 in 146 sites   11.6 %    effects > 10 perc. pnts.  (vice-versa in brackets)  P2\_Q: 13.5 (38.4) |  |
| **P2prime\_T**  9 in 146 sites   6.2 %    effects > 10 perc. pnts.  (vice-versa in brackets)  P1\_G: 41.2 (17.6) |  |
| **P2prime\_V**  20 in 146 sites   13.7 %    effects > 10 perc. pnts.  (vice-versa in brackets)  P2\_K: 16.3 (16.3)   P1\_R: 19.7 (26.3)   P3prime\_A: 18.8 (12.1) |  |
